# Alk5/Runx1 signaling mediated by extracellular vesicles promotes vascular repair in acute respiratory distress syndrome

**DOI:** 10.1186/s40169-018-0197-2

**Published:** 2018-06-22

**Authors:** Trushil Shah, Shanshan Qin, Mona Vashi, Dan N. Predescu, Niranjan Jeganathan, Cristina Bardita, Balaji Ganesh, Salvatore diBartolo, Louis F. Fogg, Robert A. Balk, Sanda A. Predescu

**Affiliations:** 1 Department of Internal Medicine, Pulmonary, Critical Care and Sleep Medicine, Rush University Medical Center, 1750W Harrison St. 1535 JS, Chicago, IL 60612 USA; 2 College of Nursing, Rush Medical College, Chicago, IL USA; 3 University of Illinois at Chicago, Chicago, IL USA; 4 Present Address: Pulmonary and Critical Care Medicine, UTSouthwestern Medical Center, Dallas, TX USA; 5 Present Address: Pulmonary, Allergy and Critical Care Medicine, University of Alabama at Birmingham, Birmingham, AL USA

**Keywords:** Acute respiratory distress syndrome, Circulatory extracellular vesicles, Runx1 isoforms, Alk5, Endothelial cell injury

## Abstract

**Background:**

Pulmonary endothelial cells’ (ECs) injury and apoptotic death are necessary and sufficient for the pathogenesis of the acute respiratory distress syndrome (ARDS), regardless of epithelial damage. Interaction of dysfunctional ECs with circulatory extracellular vesicles (EVs) holds therapeutic promise in ARDS. However, the presence in the blood of long-term ARDS survivors of EVs with a distinct phenotype compared to the EVs of non-surviving patients is not reported. With a multidisciplinary translational approach, we studied EVs from the blood of 33 patients with moderate-to-severe ARDS.

**Results:**

The EVs were isolated from the blood of ARDS and control subjects. Immunoblotting and magnetic beads immunoisolation complemented by standardized flow cytometry and nanoparticles tracking analyses identified in the ARDS patients a subset of EVs with mesenchymal stem cell (MSC) origin (CD73^+^CD105^+^Cd34^−^CD45^−^). These EVs have 4.7-fold greater counts compared to controls and comprise the transforming growth factor-beta receptor I (TβRI)/Alk5 and the Runx1 transcription factor. Time course analyses showed that the expression pattern of two Runx1 isoforms is critical for ARDS outcome: the p52 isoform shows a continuous expression, while the p66 is short-lived. A high ratio Runx1p66*/*p52 provided a survival advantage, regardless of age, sex, disease severity or length of stay in the intensive care unit. Moreover, the Runx1p66 isoform is transiently expressed by cultured human bone marrow-derived MSCs, it is released in the EVs recoverable from the conditioned media and stimulates the proliferation of lipopolysaccharide (LPS)-treated ECs. The findings are consistent with a causal effect of Runx1p66 expression on EC proliferation. Furthermore, morphological and functional assays showed that the EVs bearing the Runx1p66 enhanced junctional integrity of LPS-injured ECs and decreased lung histological severity in the LPS-treated mice.

**Conclusions:**

The expression pattern of Runx1 isoforms might be a reliable circulatory biomarker of ARDS activity and a novel determinant of the molecular mechanism for lung vascular/tissue repair and recovery after severe injury.

**Electronic supplementary material:**

The online version of this article (10.1186/s40169-018-0197-2) contains supplementary material, which is available to authorized users.

## Background

Acute respiratory distress syndrome is characterized by a consistent, recognizable pattern of lung injury; it is a life-threatening inflammatory lung condition with no drug treatment and high mortality [1–3]. The extent of repair mechanisms and outcome are very variable, and patients react differently to similar injury. If ARDS patients can survive the initial insult, lungs return to near-normal physiology except for a mild persistent reduction in diffusion capacity [4]. However, over the course of the disease, patients accumulate significant physical and cognitive disabilities [5]. Understanding the final common pathway of lung repair process might allow us to control the initial inflammation and regulate proliferation and fibrosis to achieve expedited recovery and thus, prevent the burden of physical and cognitive disabilities. Growing evidence suggests that intercellular communication of injured ECs with the EVs released in vitro by the cultured bone marrow-derived MSCs (EV_MSC_) hold significant therapeutic promise for ARDS [6]. EVs are released from a variety of cells as byproducts of cell growth, apoptosis and in response to physiologic and pathophysiologic stimuli [7]. These EVs, also known as microparticles, microvesicles, microsomes, lipid vesicles and exosomes encapsulate small portions of the subjacent cytosol, creating a heterogeneous population of phospholipid-walled vesicles [7, 8]. The EVs circulate in the blood for an unknown length of time, interact with ECs and depending on their cellular origin/cargo may have different effects on EC function [8]. The studies using EVs released by the circulating MSCs in the blood are limited [9]. The number of circulating MSCs in peripheral blood is low, but injury and inflammatory states increase it; growing evidence indicates that MSCs migrate from their specific niches (i.e., bone marrow, adipose tissue, umbilical cord), transit through the blood to the injured tissue and promote the repair process [7, 9–11].

Recently we have shown that in vivo deficiency of intersectin-1s [(ITSN); a prevalent protein of the lung tissue] triggers apoptosis of mouse lung ECs, increase in the alveolar-capillary permeability, protein-rich edema and lung injury [12]. Moreover, the cells of the vascular system release in the systemic circulation of ITSN-deficient mice a population of EVs comprising the widely expressed TβRI/Alk5. These EVs interact with the dysfunctional lung ECs, mediate the intercellular transfer of Alk5 and rescue them from apoptotic death by activation of Erk1/2 MAPK pro-survival signaling. Within 2 weeks after severe injury, lung function returned to a normal state with little evidence of prior damage, suggesting that a lung repair process was critical for the remarkable recovery [12].

Thus, we hypothesized that similar to mouse studies, ARDS patients have a subset of circulatory EVs which rescue pulmonary microvascular ECs from apoptotic death. With a multidisciplinary translational approach, we studied EVs from the blood of ARDS patients and identified a sub-population whose phenotype is different from the EVs of non-surviving patients; due to their disease-specific cargo, they provide a survival advantage to ARDS patients.

## Methods

Human lung microvascular ECs were obtained from Lonza (Walkersville, Inc., MD). The human bone marrow-derived MSCs, passage 1, were from the Institute for Regenerative Medicine, Texas A&M Health Science Center (Temple, TX).

Specific antibodies (Abs) were as follows: Alk5 rabbit Ab (Abcam; Cambridge, MA); ITSN-1 (Bethyl Laboratories, Inc., Montgomery, TX); actin and Prestige ITSN-1 Abs (Sigma-Aldrich; St. Louis, MO); TGFβRII, Runx1, Ki67, CD45, CD34, CD9, CD81, CD63, syntenin-1, mitofilin Abs (Santa Cruz Biotechnology; Santa Cruz, CA); CD31 Ab (Abbiotec, San Diego CA); phycoerythrin (PE)-conjugated CD62, CD61, CD14, CD68, CD144, CD73, CD105, CD45, CD34Abs, rabbit IgG-allophycocyanin (APC)-conjugated (Affymetrix; Santa Clara, CA) and biotin anti-human CD105 Abs (BioLegend; San Diego, CA); reporter Abs, fluorophor-conjugated, neutrAvidin-Alexa Fluor 594 and the Prolong Antifade reagent (Molecular Probes, Eugene, OR). All other reagents were purchased as follows: Spherotech nano fluorescent beads (Spherotech, Inc.; Lake Forest, IL); MagSiSta 1.0 magnetic beads (Amsbio LLC.; Cambridge MA); LPS from *Escherichia coli* 0111:B, the In Situ Cell Proliferation kit [Bromodeoxyuridine (BrdU) assay] and the protease inhibitor cocktail for mammalian cell and tissue extracts (Sigma-Aldrich; St. Louis, MO); 3-(4,5-dimethylthiazol-2-yl)-2,5-diphenyl tetrazolium bromide (MTT) Cell Proliferation Assay kit (ATCC; Manassas, VA); the bicinchoninic acid (BCA) Protein Assay Kit (Pierce; Rockford, IL).

### Research subjects

Data and sample collection was done following the Rush University Medical Center (RUMC) Institutional Review Board (IRB), using an approved protocol (IRB# 14030705-IRB01) for investigational use of un-used diseased blood samples, drawn for routine medical care. Blood was collected from 33 patients admitted to RUMC ICU; all patients included, 18 years and older, were identified within 24 h of diagnosis and met moderate-to-severe ARDS criteria per “The Berlin Definition of ARDS” [13]. Age < 18 years, patients with isolated left heart failure and active malignancy and patients who received immunosuppressant or chemotherapy during ARDS hospitalization were excluded. Age, sex, race, Acute Physiology and Chronic Health Evaluation II (APACHE II), Simplified Acute Physiology Score II (SAPS II), Sequential Organ Failure Assessment (SOFA) score, Lung Injury Score (LIS), P/F (PaO_2_/FiO_2_) ratio on the day of ARDS diagnosis, cause of ARDS, ventilator-free days, extracorporeal membrane oxygenation (ECMO), and length of stay were collected. Mortality from all causes was recorded at day 100. A detailed clinical data set is included in Additional file 1: Table S1.

Pathological slides (paraffin-embedded lung tissue) of five ARDS subjects and three non-disease controls (ND-Ctrl) identified from autopsy files were provided by the Department of Pathology, RUMC. Frozen lung tissue, normal and ARDS, was obtained from the National Disease Research Interchange. Clinical diagnosis, underlying conditions, and other pertinent clinical and laboratory data were reviewed.

### Animals

CD1 male mice, 6–8 weeks old, 20–25 g weight, from Jackson Laboratory (Bar Harbor, ME), kept under standardized housing and feeding conditions were used. All mouse studies were approved and performed under the guidelines of RUMC Institutional Animal Care and Use Committee. The experiments were done under anesthesia [ketamine (60 mg/kg), acepromazine (2.5 mg/kg) and xylazine (2.5 mg/kg)] in 0.1–0.2 ml phosphate buffered saline (PBS)]. Three to five mice per experimental condition (wt-mice, LPS- and LPS ± EVs-treated mice) were used; all experiments were repeated at least three times. No mouse mortality occurred during the study.

### Lung histology, immunohistochemistry (IHC) and morphometric analysis

Mouse lungs were inflated with 1% low-melting-point agarose in 10% formalin at a constant pressure of 25 cm H_2_O, allowing for homogenous expansion of lung parenchyma, and then fixed in 4% paraformaldehyde for 48 h and paraffin-embedded [12]. Thin sections (4–5 μm), cut longitudinally, were stained with hematoxylin/eosin (H&E). Images were acquired with a 20× lens using a Zeiss AxioImager M1 motorized upright microscope equipped with AxioCam ICc1 R3 RGB color digital camera (Carl Zeiss MicroImaging, Inc., Thornwood, NY). Quantification of perivascular cuffing area was performed on small and medium-sized (20 μm ≥ diameter ≤ 100 μm) blood vessels using the NIH ImageJ software version 1.8.0_112 as described previously [14]. A minimum of 25 vessels per section was used (three sections/mouse, 3–5 mice/experimental condition). All experiments were performed at least three times with reproducible results.

IHC on paraffin-embedded human lung tissue sections was performed using the Prestige ITSN-1 Ab (C-terminal epitope; the only commercially available Ab efficient in IHC), CD31 Ab and Ki67 Abs. All were followed by the appropriate Alexa Fluor 488- or Alexa Fluor 594-conjugated reporters as previously described [12]. CD31 and Ki67 Abs were used at 1:200 dilution in 0.1% BSA in PBS, whereas ITSN-1 and was used at 1:100 dilution.

### EVs isolation and standardization

EVs were isolated from the blood of ARDS patients (EV_ARDS_) and control healthy subjects (EV_Ctrl_). Whole blood was subjected to a first centrifugation [1.5×*g*; (3000 rpm) for 15 min, at 4 °C; Eppendorf microfuge, 5702R], to obtain the platelet-free plasma, that was then subjected to ultracentrifugation (Beckman Coulter, Optima™ MAX-XP, TLA-55 fixed angle rotor, 45-degree angle) at 79,700×*g*; (36,000 rpm), for 2 h, at 4 °C [15]. The EVs pellet was washed (3 × 10 min) in sterile PBS, subjected to ultracentrifugation as above and resuspended in 200 μl sterile PBS. To minimize potential changes, if any, in EVs’ stability, only freshly prepared EVs were used for functional and morphological studies. EVs stored in liquid N_2_ were used only for biochemical studies. To achieve high scientific rigor regarding reproducibility, the EVs were standardized according to the guidelines established by the International Society of Extracellular Vesicles for EVs isolation and analyses [16, 17]. We performed the following: (i) use of plasma for EVs retrieval; (ii) venipuncture and 0.109 M Na citrate as coagulant; (iii) room temperature (RT) for blood storage before first centrifugation; (iv) EVs isolation within 2 h of blood collection; (v) ultracentrifugation and immunobead magnetic separation for EVs isolation/enrichment, (vi) flow cytometry and calibrated/counted beads to establish the EVs’ cellular origin and counts, (vii) collection of blood at the same time of the circadian day. Other key standardization factors specific to this study are: (i) constant blood volume to isolate EVs, (ii) constant 200 μl PBS to resuspend the EVs pellet, (iii) establish the Runx1p66/p52 ratio in the EVs isolated from the blood samples collected in week 2 of ICU stay (days 7–14), when the patients are in the sub-acute, proliferative phase of the disease, characterized by repair of the damaged alveoli and restoration of the barrier function [18].

### ECs culture, LPS treatment, and EVs exposure

ECs, passages 3–5, were grown in Endothelial Basal Medium-2 and medium 199-supplemented with 20% fetal bovine serum (FBS) as previously described [19]. To mimic the inflammatory EC dysfunction, the ECs were treated with 1 μg/ml LPS for 6 h (EC_LPS_). Following LPS treatment, EC_LPS_ were exposed to three doses of EVs for 30 h, with 1 μg/ml LPS still present in the growth media.

### Human MSCs culture and isolation of EV_MSC_

Cells were used for the experimental protocols between passages 2 and 4. The MSCs were cultured in α-Minimum Essential Medium without ribonucleosides and deoxyribonucleosides containing 2 mM l-glutamine, 10% FBS (Atlanta Biochemicals, Inc., Flowery Branch, GA), 100 units/ml penicillin and 100 mg/ml streptomycin (Thermo Fisher Scientific, Hanover Park IL), in a humidified incubator at 5% CO_2_ and 37 °C under sterile conditions [20, 21].

EV_MSC_ were obtained from the conditioned medium of MSCs at different time points of culture. MSCs were incubated for 24 h in medium depleted of FBS-derived EVs [22]. The conditioned was centrifuged at 1.5×*g*; (3000 rpm), for 15 min to remove cellular debris, then at 79,700 × g; (28,000 rpm), Beckman Coulter XL-90 ultracentrifuge, 70 Ti rotor, for 2 h at 4 °C. EV_MSC_ pellet was washed in PBS and subjected to a second ultracentrifugation. The EV_MSC_ were resuspended in sterile PBS according to the count of MSCs, usually 500 μl sterile PBS for 2 × 10^6^ cells. EV_MSC_ were lysed for 1 h, at 4 °C in 50 mM Tris–HCl, pH 8.0, 150 mM NaCl, 1% NP-40 and protease inhibitor cocktail, used as per manufacturer’s instructions. The protein content of the EV_MSC_ was quantified by the BCA assay with bovine serum albumin as standard.

### Cell proliferation assays

#### BrdU assay

Cells were grown on coverslips for 48 h. BrdU incorporation was performed as described previously [12, 23]. Briefly, cells were incubated in culture medium containing 10 μM BrdU Labeling Solution for 6 h at 37 °C. Cells were then washed with PBS, fixed and the DNA denatured followed by incubation with BrdU-FLUOS Ab (45 min, 37 °C) in a humid chamber. Cells were again washed with PBS and the coverslips were mounted using the Prolong Antifade kit. The BrdU positive cells were counted on high power field images, and data were expressed as the number of BrdU positive cells per 50 high power fields.

#### MTT assay

Triplicate aliquots of ECs (10^6^ cells suspended in 100 μl complete EC medium) were seeded onto a 96-well plate, and serial dilutions were prepared in EC medium. Cells, cultured for 48 h, were subjected to the LPS and EVs exposure as described above, followed by addition of 10 μl MTT Reagent to each sample. After 5 h incubation, 100 μl of detergent was added to each well; the plate was covered and kept in the dark at RT overnight. Absorbance was measured at 595 nm (OD^595^) in a microtiter plate reader on the following day. Parallel triplicate experiments using non-treated ECs were performed, cells were counted using a hemocytometer, and a growth curve was generated to relate the OD^595^ values to the cell number per well [24].

### Protein extraction and Western blot (WB) analyses

EV_ARDS_ and EV_Ctrl_ were lysed, and protein concentration was determined as described for EV_MSC_. EVs lysates (individual samples) were analyzed for Alk5, TβRII and Runx1 protein content by WB via Alk5 (1:500), TβRII (1:500), Runx-1, CD9, CD81, CD63, syntenin-1, mitofilin (1:1000) Abs followed by the appropriate horseradish peroxidase-conjugated reporters [15]. WB analyses of EV_ARDS_ and EV_Ctrl_ lysates were normalized to equivalent μl blood or the total protein, as specified in the text. Actin cannot be used as loading control, as there is no evidence that EVs contain actin.

### Flow cytometry and EVs counting

Total number/percentages of EVs were determined by standardized flow cytometry (Beckman Coulter Gallios flow cytometer with Kaluza G software for acquisition and Kaluza1.3 for analysis; [15, 25]), where the number of EVs was correlated to a fixed number of counting beads. EVs gating was accomplished by preliminary standardization experiments using Spherotech nano fluorescent size standard beads. Data are presented as dot plot and histograms and the results of data analysis as average % of total gated events (at least 10,000 events/sample) ± SEM. Percentage labeled EVs was determined by comparing with unstained control EVs, with gates set to exclude beads, background as determined by a buffer alone sample, as well as aggregates. Isotype-matched Abs served as control.

#### Nanoparticle tracking analysis (NTA)

The size distribution and concentrations of EV_Ctrl_ and EV_ARDS_ preparations were determined by NTA [26, 27], using a NanoSight NS 300 instrument (Malvern Instruments Limited; Malvern, Worcestershire, UK) and statistical analysis was performed for particles with diameter lower than 300 nm. Aliquots of each EV_Ctrl_ and EV_ARDS_ sample were diluted 1:100 in sterile PBS and placed in 1 ml syringes. Five 60 s frames were captured for each sample to ensure accurate quantitation of the sizes and number of EVs. The data were averaged to determine the distribution and concentration of particles in each sample using the Nanosight NTA software Version 3.2 Dev Build 3.2.16.

### Immunobead magnetic separation

Immunobead magnetic separation of Alk5-positive EVs was achieved in three steps as follows: EV_ARDS_ were labeled with Alk5 polyclonal Ab [15]; then a biotin-conjugated IgG secondary Ab was used as a bridge to allow the binding of the MagSi-STA 1.0 magnetic silica beads to the EV_ARDS_. All three steps were followed by successive washings in PBS followed by ultracentrifugation as above. Isolation of CD105-positive EVs was achieved via biotinylated Abs using a similar approach. Aliquots of EVs bound to the magnetic beads were analyzed by SDS PAGE and WB for Alk5, Runx1, CD105, CD34 and CD45 immunoreactivity. EV_ARDS_ preparations were normalized to the blood volume used for isolation. Incubations of the EVs with the biotin-conjugated IgG secondary Ab and the MagSi-STA 1.0 magnetic silica beads by omitting the first Ab were used for controls.

### EVs imaging

EVs were labeled with biotin/neutrAvidin–Alexa Fluor 594 or double labeled with Alk5 rabbit Ab/anti-rabbit IgG Alexa Fluor 488 and biotin/neutrAvidin–Alexa Fluor 594 reporters [15]. Final pellets were resuspended in PBS and fixed in 1% paraformaldehyde; aliquots were mounted on glass slides with Prolong Antifade Reagent. Isotype-matched IgG was used as a control. EVs were examined and photographed using a Zeiss AxioImager M1 microscope.

### Power and statistical analysis

The human analysis used a Kaplan–Meier test (SPSS software, Version 22), to determine if the survival is longer among the Runx1p66 immunoreactive subjects. An effect size of d = 1.20 was obtained [28], which may be a little optimistic. Thus, assuming a more conservative effect of d = 0.80 (Cohen’s estimation of a ‘large’ effect), for our sample size of 29 ARDS patients (four patients out of the total of 33, were used only for flow cytometry; no Runx1 expression pattern was analyzed), a one-tailed alpha of 0.05, a power of 0.97 are obtained.

A standard heterogeneous *t* test was used to compare data between groups.

To look for differences in the two groups as shown in Table 3, we used χ^2^ test for differences in mortality and sex and utilized student *t* test to look for differences in age, P/F ratios, APACHE II, SAPS II, SOFA score, and LIS.

## Results

### Human ARDS lung tissue is deficient of ITSN and shows limited EC proliferation

**Fig. 1:**
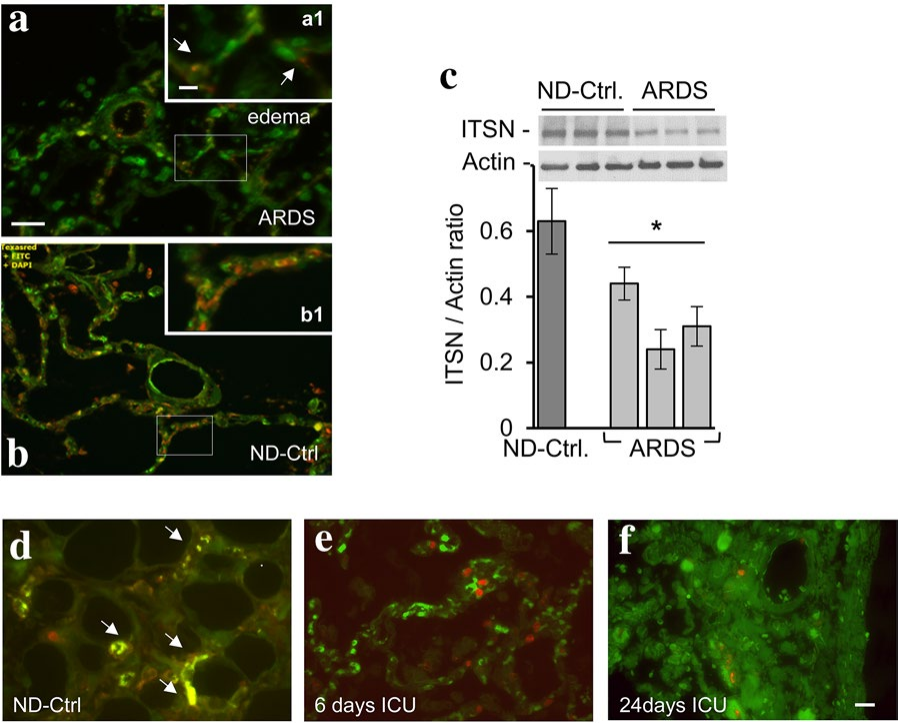
Human ARDS lung tissue is deficient of ITSN and shows limited EC proliferation. **a**, **b** Representative ITSN-Alexa Fluor 594/CD31-Alexa Fluor 488 IHC of lung sections of ARDS and ND-Ctrl specimens, respectively; insets are higher magnifications of boxed areas. The arrowheads in **a** indicate a lung vessel with perivascular edema. Arrows in **a**, inset indicate the limited co-localization ITSN/CD31. Bars: 60 μm (**a**, **b**); 100 μm (insets). **c** Total protein extracted from ND-Ctrl and ARDS frozen lung tissues was analyzed by SDS PAGE (50 μg/lane) and WB with ITSN Ab. Actin served as loading control. The graph in **c** shows the average of ITSN/Actin ratio ± SD as determined by densitometric analyses of representative blots. *p < 0.01 vs. ND-Ctrl. n = 3. **d** Representative Ki67-Alexa Fluor 594/CD31-Alexa Fluor 488 IHC of lung sections of ND-Ctrl, and 2 ARDS specimens, a 55 year old male (**e**), and a 65 year old male (**f**); the number of days in ICU are indicated. Arrows in **d** indicate the co-localization CD31/Ki67. Sepsis was the cause of ARDS. Five ARDS and three ND-Ctrl samples were analyzed. Bar: 50 μm. n = 3 experiments

As chronic deficiency of ITSN in a murine model of acute lung injury (ALI)/ARDS triggers a repair process characterized by EC proliferation and vascular remodeling [12], we investigated whether ITSN deficiency is a feature of ARDS human lung tissue. ITSN/CD31 IHC was used to study the cellular distribution of ITSN in the lung tissue of ARDS and ND-Ctrl specimens. ITSN/Alexa Fluor 594 staining is limited in the ARDS specimen (55 years old, male; pneumonia; Fig. 1a, inset, arrows). The typical ARDS morphology with tissue congestion, inflammatory infiltrates, and edema (Fig. 1a, arrowheads) is noticeable. The ND-Ctrl shows significant ITSN immunoreactivity and co-localization with the EC marker CD31 (Fig. 1b, inset). For biochemical evaluation of ITSN expression, total protein was extracted from frozen human ND-Ctrl lung (62 years old male, myocardial infarction) and ARDS tissue (36 years old male, pneumonia), from three different locations. WB followed by densitometry indicate that expression of ITSN protein is decreased 40–70% in ARDS samples compared to ND-Ctrl (Fig. 1c). This finding is highly relevant to ECs of the lung, as ECs represent about 50% of the lung tissue [29].

As ECs proliferation and increased microvascular density were critical for the repair process of murine lung injury [12], we next used lung autopsy slides of 5 ARDS patients who survived 6–24 days in ICU and subjected them to dual Ki67 (proliferation marker)/CD31 IHC to investigate the extent of EC proliferation. ARDS specimens show limited ECs proliferation (Fig. 1e, f), most likely the cause of the poor lung architecture and increased fibrosis in these patients. Ki67/CD31 co-localization on a lung section of a ND-Ctrl specimen is shown for comparison (Fig. 1d, arrows).

### The systemic circulation of ARDS patients contains elevated levels of EV_ARDS_ immunoreactive to TβRI/Alk5

**Fig. 2:**
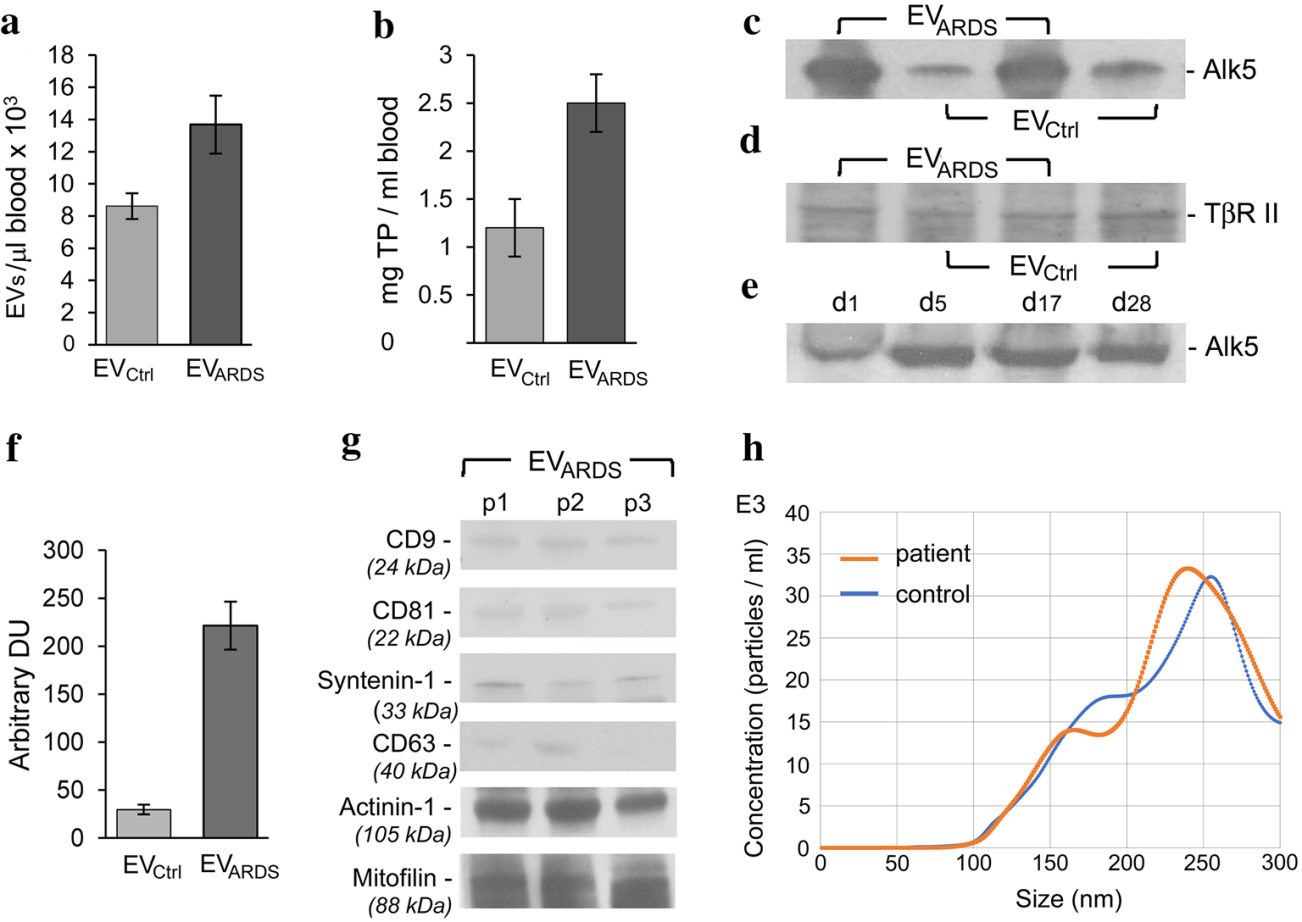
ARDS subjects show increased counts of circulatory EV_ARDS_ immunoreactive to TβRI/Alk5. **a** The mean count for EVs isolated from the blood of the ARDS patients (EV_ARDS_) was 13,680 ± 1811, with a range 11,000–17,500; Nine EV_ARDS_ preparations from nine different ARDS patients were used. The EV_Ctrl_ mean count indicated 8615 ± 768, with a range 7700–9200; three preparations from the blood of three different control subjects were used. Data are normalized per 1 μl blood. Three independent experiments were performed. **b** Amount of total protein in the EV_ARDS_ pellet and EV_Ctrl_ pellet, normalized to 1 ml blood. Data are plotted as mean ± SD; *p < 0.05 vs. controls. **c**, **d** WB of Alk5 and TβRII protein expression in two EV_ARDS_ and two EV_Ctrl_ lysates, normalized to equivalent μl blood; n = 9. Actin cannot be used as loading control, as there is no evidence that the EVs contain actin. **e** Time course analysis of Alk5 in EV_ARDS_ in one of the above ARDS cases; n = 3. Day 1 is first day in ICU. **f** Densitometric analysis of Alk5 protein expression in the EV_Ctrl and_ EV_ARDS_ lysates. Data are plotted as mean ± SD; *p < 0.05 vs. controls. **g** Size distribution and concentration of exosomes and smaller than 300 nm diameter microparticles in the isolated EV_Ctrl_ and EV_ARDS_ preparations, measured by NTA; n = 3. **h** Representative WB analyses of EV_ARDS_ fractions of three different patients using Abs specific for CD9, CD81, syntenin-1, CD63, actinin-1 and mitofilin. Actinin-1 and mitofilin, considered specific markers for the non-exosomal microvesicles are enriched in the EV_ARDS_ preparations; n = 3

EV_ARDS_ and EV_Ctrl_ were isolated from the blood of ARDS and control subjects within less than 2 h of blood withdrawal. Accurate data for EVs concentration were obtained by standardized flow cytometry [25]. EVs’ count indicated mean values of 13,680 EV_ARDS_/1 μl blood and 8615 EV_Ctrl_/1 μl blood, Fig. 2a. The EVs were also quantified by comparing the total protein amounts in the EV_ARDS_ and EV_Ctrl_ pellets. The protein content of EV_ARDS_ is ~ 2-fold higher compared to the EV_Ctrl_, Fig. 2b, an expected higher ratio given the aggregates content, excluded from the gated flow cytometry count. This ratio was recorded regardless of the timing of blood collection from all ARDS patients studied, consistent with reports of elevated levels of circulating EVs in disease settings [9, 11, 30]. As ARDS lung tissue is deficient of ITSN, we investigated whether EV_ARDS_ contain the TβRI/Alk5, similar to the EVs released in the circulation of ITSN-deficient mice. EV_ARDS_ and EV_Ctrl_ lysates were analyzed for their Alk5 content by WB, Fig. 2c. Alk5 was found in both EV_Ctrl_ and EV_ARDS_. However, as indicated by densitometric analyses of representative blots, Fig. 2f, Alk5 expression was sevenfold greater in EV_ARDS_ compared to EV_Ctrl_, suggestive of a greater expression of Alk5 in EV_ARDS_ compared to the EV_Ctrl_. Since TβRI signals through a heteromeric TβRI/TβRII complex and since the EVs of ITSN-deficient mice were immunoreactive for the TβRII [15], we also analyzed the EV_ARDS_ for their TβRII content by WB. EV_ARDS_ were immunoreactive to the TβRII Ab, Fig. 2d, suggesting that the human EVs, bear the Alk5/TβRII heteromers. Furthermore, time course analyses of Alk5 protein expression in the EV_ARDS_ from different ARDS subjects at several time points of ICU stay indicate no significant changes in Alk5 from day 5 to day 28 of ICU stay, Fig. 2e, the time interval used to collect blood and isolate EV_ARDS_.

As the size ranges of EVs, from exosomes (diameter 40–120 nm) to microparticles, diameter 200–1000 nm [31], cannot be considered absolute and given the unfeasibility to isolate a pure microparticle fraction, exclusively [16], we also evaluated the exosomal content by NTA approach (Fig. 2g). No discernible peak was detected for particle sizes of 40–120 nm. We also evaluated the purity of the isolated EVs fractions by WB analyses using specific Abs against CD9, CD81, syntenin-1 and CD63, reported to be enriched in exosomes, and actinin-1 and mitofilin enriched in non-exosomal EVs populations [22]. As shown in Fig. 2h, the EVs fractions of three different ARDS patients showed scarcely detectable immunoreactivity for exosomal markers and strong immunoreactivity for the non-exosomal EVs, consistent with an enriched non-exosomal fraction of EV_ARDS_. Thus, the isolation procedure applied indicates that the exosome and microparticle fractions are distinct in both size and biochemical makeup.

It is widely accepted that it is difficult to distinguish between two subpopulations of EVs based only on the size and protein composition [22]. Since a precise definition for exosomes and microparticles remains to be resolved, we refer to the population of membrane vesicles isolated according to our experimental protocol (79,700 × g, 2 h), as EVs.

### EV_ARDS_Alk5 sub-population is derived from the MSCs

**Fig. 3:**
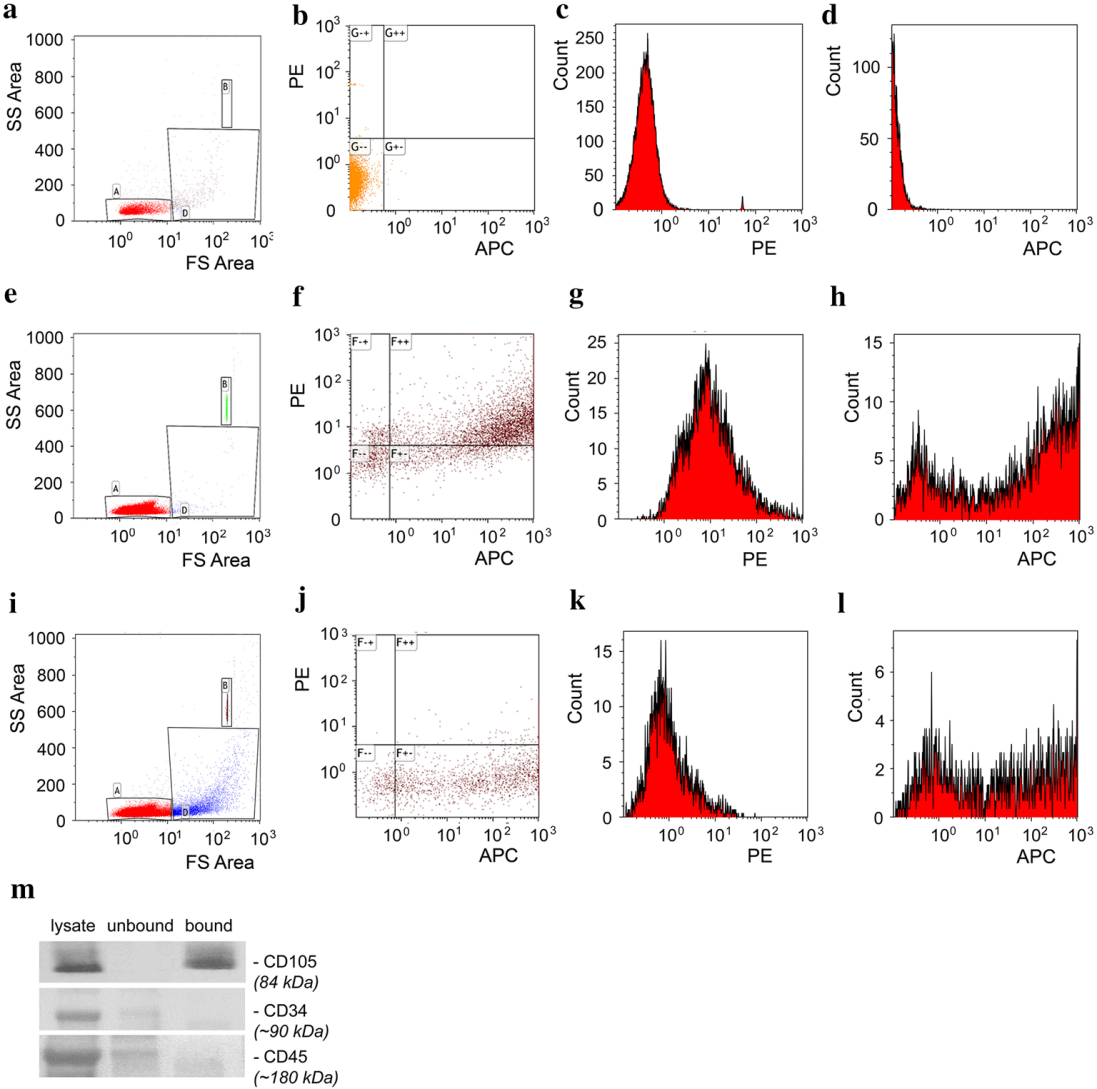
EV_ARDS_Alk5 sub-population is derived from the MSCs. Unstained EVs were used as controls to determine fluorescence background as well as for gating purposes. **a** Representative forward and side scatter plot for unstained EVs. Background fluorescence in the PE and APC channels for the unstained samples shown as dot plot (**b**) and individual histograms (**c**, **d**). Similar, representative plot of EVs stained with Alk5-APC/CD105-PE Abs (**f**) to analyze the co-expression of these markers on the EV_ARDS_ (**e**, Gate D), together with the individual histograms for CD105-PE (**g**) and Alk5-APC (**h**). Representative plot of EVs stained with Alk5-APC/CD73-PE Abs (**j**) to analyze the co-expression of these markers on the EV_ARDS_ (**i**, Gate D), along with the histograms for CD73-PE (**k**) and Alk5-APC (**l**) are also shown. n = 9 EV_ARDS_ and 5 EV_Ctrl_ isolates. **m** Representative immunoisolation of CD105^+^ EV_ARDS_ Alk5 via biotin-conjugated CD105 Ab and streptavidin magnetic beads incubation. Lysates of EV_ARDS_, bound and unbound EV_ARDS_ fractions were analyzed by SDS PAGE and WB. No immunoreactivity to CD45 and CD34 is detected. n = 6 EVs isolates/experimental condition, in three independent experiments

Labeling of EV_ARDS_ using allophycocyanin-Alk5, phycoerythrin- and pacific blue-conjugated cell surface markers, followed by flow cytometry analyses of the mean values/percentages recorded, indicate: (i) a high content (> 50%) of EV_ARDS_ immunoreactive to Alk5 Ab, (ii) immunoreactivity of EV_ARDS_Alk5 to CD105 and CD73 Abs, suggestive of MSC-origin, (iii) 4.7-fold increase in the count of EV_ARDS_ of MSC-origin compared to EV_Ctrl_, as determined by tri-color labeling of EVs (Table 1). For data analysis, scatter and fluorescence noise was determined by running double filtered buffer alone, and a gate was drawn over the background noise to exclude those events (Fig. 3a, Gate A). Representative forward (FS) and side scatter (SS) plot for unstained EV_ARDS_ and background fluorescence in PE and APC channels for the unstained samples are shown as dot plot (b) and individual histograms (c, d). Samples of isolated EVs (Gate D) and 2 μm beads (Gate B) were run, to determine approximate sizing of the EVs relative to the beads as well as to determine background fluorescence (Fig. 3e, i). The majority of EVs were found to be around 1 μm in size (Gate D). Next, EV_ARDS_ stained with Alk5-APC/CD105-PE Abs were analyzed by flow cytometry to evaluate the co-expression of Alk5 and CD105 on the EV_ARDS_ (Fig. 3e, Gate D). A representative plot of double stained EV_ARDS_ (Fig. 3f) and the individual histograms for PE-CD105 (Fig. 3g) and APC-Alk5 (Fig. 3h) are shown. Similarly, EV_ARDS_ co-stained with Alk5-APC/CD73-PE Abs were analyzed by flow cytometry to evaluate the co-expression of Alk5 and CD73 on the EV_ARDS_ (Fig. 3i, Gate D). A representative flow cytometry plot of Alk5-APC/CD73-PE-labelled EV_ARDS_ is shown in Fig. 3j. The individual histograms for CD73-PE (Fig. 3k) and Alk5-APC (Fig. 3l) are shown.

**Table 1:** A subset of EV_ARDS_ has mesenchymal stem cell (MSC)-origin and shows significant increase in the ARDS subjects compared to controls

Variable (EVs/μl)	Mean		Fold increase
	EV_Ctrl_	EV_ARDS_	
Alk5	3446 (40%)	7200 (53%)	2.1
Alk5/CD73	53 (0.6%)	294 (2.1%)	5.6
Alk5/CD105	478 (5.5%)	1008 (7.4%)	2.1
Alk5/CD62	632 (7.3%)	1021 (7.5%)	1.6
Alk5/CD68	534 (6.2%)	1123 (8.2%)	2.1
Alk5/CD31	432 (5.0%)	782 (5.7%)	1.8
Alk5/CD73/CD105	49 (0.57%)	234 (1.7%)	4.8

Mean numbers/percentages of EV_Ctrl_ and EV_ARDS_ co-labeled with allophycocyanin (APC)-Alk5, phycoerythrin (PE) and pacific blue-conjugated cell surface markers. We used CD62 for platelets [59], CD68 as monocytes/macrophage markers [60, 61], CD31 for ECs [62, 63] and CD73 and CD105 for MSCs [32]. Isotype matched Abs served as control; n = 7 EVs isolates/experimental condition, in three independent experiments

MSCs origin of EV_ARDS_ was also confirmed by no detectable immunoreactivity to the hematopoietic markers CD45/CD34 [32], as shown by magnetic bead immunoisolation of CD105-positive EV_ARDS_ followed by WB (Fig. 3m). As the cellular origin of EVs is a defining factor for the magnitude of the physiological effect elicited [33], and considering the 4.7-fold increase in the count of the EV_ARDS_ of MSC origin compared to controls, the most significant increase compared to the EV_ARDS_ of other cellular origins identified, we focused on this disease specific subset of EV_ARDS_.

### Runx1p66 isoform provides a survival benefit to ARDS patients

**Fig. 4:**
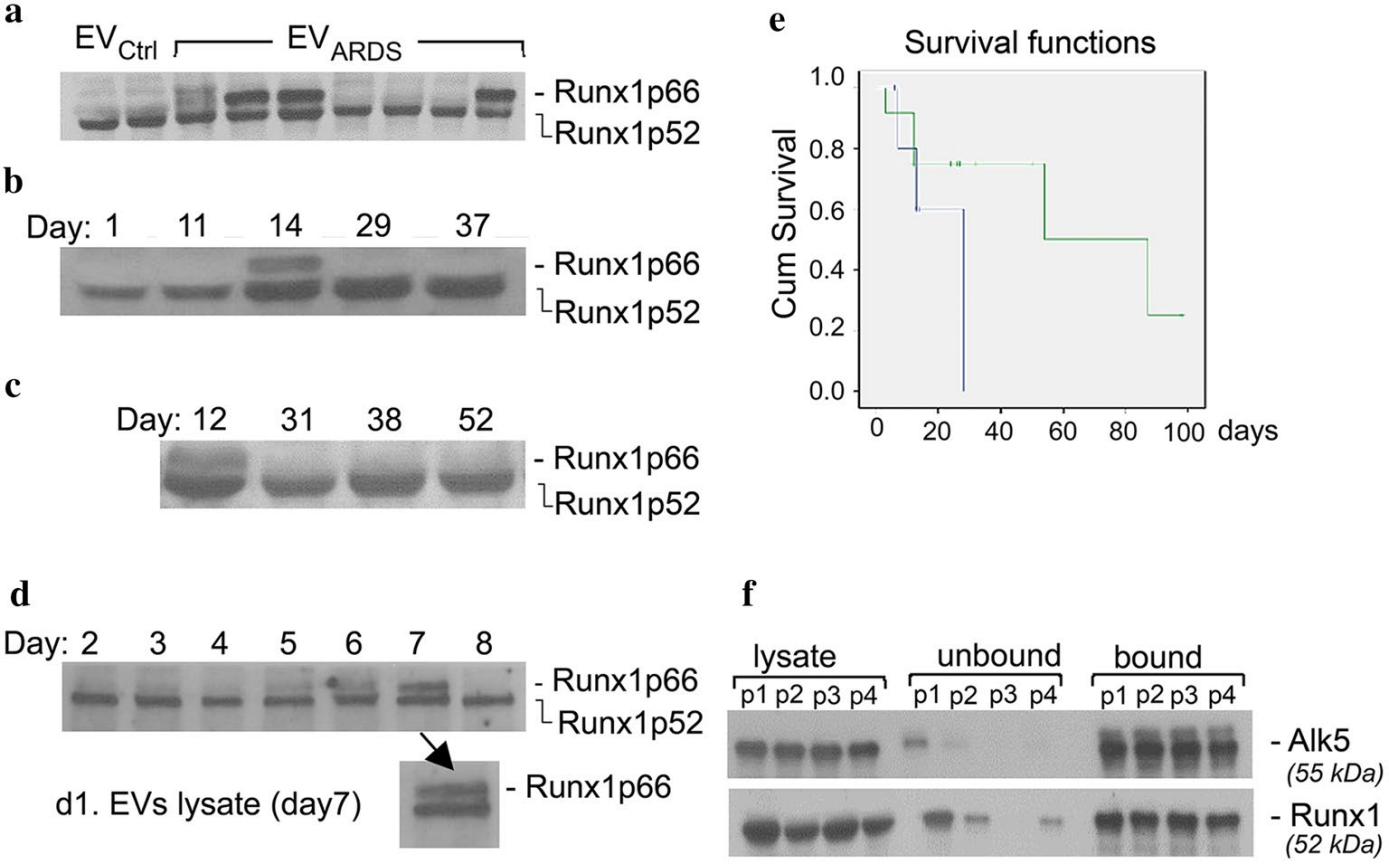
Runx1p66 protein expression is short-lived and provides a survival advantage. **a** Representative Western blot of Runx1 protein expression in seven EV_ARDS_ and two EV_Ctrl_ preparations. Normalization was achieved to the volume of blood used (typically 1.5 ml) for EVs isolation. **b** Time course analysis of Runx1 in EV_ARDS_ of a long-term ARDS survivor (S6) and panel **c**, a non-surviving ARDS patient (S12). Expression of Runx1p52 gradually increases from day 1 to day 37 in ICU in the EV_ARDS_ of long-term survivor. Even if the number of blood samples available for later time points assessed was lower due to death of some patients, the time course analysis data are representative for at least three long-term survivors and three non-surviving ARDS patients. **d** Western blot of Runx1 expression in lysates of cultured human bone marrow-derived MSCs (d2–d8) as well as in lysates of the EV_MSC_ released at d7 (d1). 30 μg protein/lane; n = 3. **e** The Kaplan–Meier statistic shows the improved survival for the EV_ARDS_Alk5Runx1p66 patients. Out of 33 ARDS patients included in the study, only 29 patients (multiple samples) were used to investigate the Runx1 isoform content and the Runxp66/p52 ratio. **f** Four EV_ARDS_ preparations (p1–p4; isolated from 1.8 ml of blood) were labeled with a biotinylated Alk5 rat Ab followed by streptavidin-conjugated magnetic beads. An isotype matched IgG for Alk5 was used as control. Aliquots of EV_ARDS_ lysates, unbound and bound fractions were analyzed by SDS PAGE and Western blot with Alk5 and Runx1 Abs. EV_ARDS_ are immunoreactive to Runx1p52 isoform only as the blood samples used belong to either non-surviving ARDS subjects or to surviving ARDS subjects, but collected in week 1 of ICU stay, when the EV_ARDS_ do not show Runx1p66 immunoreactivity. n = 9 different ARDS patients; Three independent experiments were performed

Given the involvement of Runx1 transcription factor in angiogenesis and MSCs proliferation [34] and our findings that lung tissue of non-surviving ARDS patients shows deficient EC proliferation, we investigated whether the EV_ARDS_ contain Runx1 by WB analysis. Two Runx1 isoforms were detected—a 52-kDa protein present in all EV_ARDS_ as well as in EV_Ctrl_, and a less characterized 66-kDa isoform [35], present only in some EV_ARDS_ samples, Fig. 4a. This observation is of particular interest, as out of 29 samples analyzed, 20 samples show Runx1p66 immunoreactivity; 14 out of these 20 EV_ARDS_ samples also display a higher than 0.5 Runx1p66/p52 ratio and belong to ARDS patients still alive, Table 2. Three samples (S5, S14, S35) belong to ARDS patients that died despite a high Runx1p66/p52 ratio (> 0.5) due to special circumstances, as described in Table 2 (legend), while the remaining three samples (S11, S12, S15) have a low Runx1p66/p52 ratio (< 0.5) and also belong to non-surviving ARDS patients. Other 4 EV_ARDS_ samples (S7, S8, S20, and S22) with no Runx1p66 immunoreactivity belong to non-surviving ARDS patients, as well. Notably, the last 5 EV_ARDS_ samples belong to long-term ARDS survivors that spent on average only 12.4 days in ICU; these EV_ARDS_ show no Runx1p66, Table 2, raising the possibility that the circulatory EV_ARDS_Runx1p66 might be needed only when the endogenous lung MSCs [36, 37] and thus, the ability of lung tissue to repair, a hallmark of the normal course of recovery from ALI in some patients [38], are not sufficient. The ratio Runx1p66*/*p52 was established in all cases in EV_ARDS_ isolated from blood samples collected in week 2 of ICU stay, a key standardization factor for patients’ disease stage. Histologically, days 7–14 are recognized as the sub-acute, proliferative ARDS phase, characterized by lung cell proliferation and attempts of repairing the damaged alveoli and restoration of the barrier function [18]. These observations strongly suggest that expression of Runx1p66 and a high Runx1p66*/*p52 ratio provide a survival benefit to ARDS patients. It seems that despite the heterogeneous etiology of ARDS and various treatment/medication received, concurrent illness, mechanical ventilation, active inflammation, etc., the transient expression of Runx1p66 and a high Runx1p66/p52 ratio are part of a common pathway of lung response leading to ARDS resolution.

**Table 2:** Expression of Runx1 isoforms in EV_ARDS_ and ARDS outcome

ARDS sample	Runx1^*p66*^ expression	p66/p52 ratio	ICU length of stay (days	ARDS outcome
S2; S4; S5*; S6; S10; S13; S14*; S17; S18; S25; S26, S28; S31; S32; S33; S34; S35*;	Yes	High	28	14 long-term survivors 3 deceased*
S11; S12; S15	Yes	Low (< 0.5)	51	All deceased
S3; S9; S19; S23; S27;	No	–	12.4	All alive (the endogenous lung ability to repair is sufficient for recovery)
S7; S8; S20; S22	No	–	14.5	All deceased (the endogenous lung ability to repair not sufficient for recovery and no Runx1^p66^ expression)

29 EV_ARDS_ preparations and 3 EV_Ctrl_ were lysed and subjected to WB analyses with Runx1 Ab. Runx1 immunoreactivity was quantified by densitometry (NIH ImageJ) and used to establish the Runx1p66/p52 ratio. EV_ARDS_ samples S5*, S14* and S35* illustrate special situations of three ARDS patients that died despite their high Runx1p66/p52 ratio due to other complications (S5) or removal of life support at family’s request (S14, S35)

We also performed a time course analyses of Runx1 expression in EV_ARDS_ of long-term ARDS survivors, Fig. 4b, and non-survivors, Fig. 4c; Runx1p52 is detected at each time point analyzed, while Runx1p66 is detected only at day 14 in ICU for the ARDS survivor, in the case shown. This transient expression of Runx1p66 was also detected in cultured human bone marrow-derived MSCs, Fig. 4d. Briefly, we analyzed cultures of human bone marrow-derived MSCs, passage 2–3, for their Runx1 expression, each day starting at about 5% confluence (day 2) and after the cultures had expanded to about 80% confluence (day 8). Runx1p52 was expressed starting at day 2 at each time point analyzed, while Runx1p66 was transiently expressed (days 6 and 7), and released in the EVs recovered from the growth medium.

We then analyzed the ARDS patients and their EV_ARDS_′ Runx1p66 and p52 isoform content regarding to the severity of the disease, demographics and mortality, Table 3. Only 3/17 patients with a high ratio (> 0.5) died as compared to 7/12 patients who had a low ratio (< 0.5) or Runx1p66 negative. This difference in mortality was statistically significant by Chi square test (χ^2^ = 5.15, p = 0.023). The likelihood ratio is 5.218. Furthermore, we compared these two groups for differences in age, sex, SAPS II score, APACHE II score, SOFA score, LIS and P/F ratio at the onset of ARDS and ICU length of stay. None of these were statistically significant, but patients with high Runx1p66/p52 ratio tended to be younger (mean age 41.76 ± 6.9 vs. 50.25 ± 9.4). Despite the relatively small sample size of this study, there is no question that the survival/censoring times for participants with this Runx1p66 isoform was much larger than it was for those without it; the patients are censored as the total survival time cannot be accurately determined for all participants [39]. Combining mortality and censored times, the mean time for the Runx1p66 group is 32.10 days (sd = 24.35) while the mean time for the Runx1 p66-negative group was 12.89 days (sd = 6.37). A simple, heterogeneous t-test between the two groups is statistically significant (t = 3.29, df = 23.90, p = 0.003).

**Table 3:** Comparison for baseline severity of disease, demographics and mortality between patients with Runx1p66/p52 high ratio vs. patients Runx1p66 negative or low Runx1p66/p52 ratio

	Runx1 p66/p52 high ratio	Low Runx1 p66/p52 ratio or p66 negative	p value
No.	17	12	–
Mortality	3 (17.6%)	7 (58.33)	0.023 (OR = 5.15)
Age	41.76 ± 6.4	50.25 ± 9.4	0.142
Sex	10F/7M	5F/7M	0.362
SAPS II	47.23 ± 6.9	53.33 ± 7.25	0.254
APACHE II	23 ± 3	25.5 ± 4.35	0.349
SOFA	10.94 ± 1.58	13.08 ± 2.18	0.124
Lung Injury Score	2.91 ± 0.33	3.09 ± 0.49	0.574
P/F ratio at onset of ARDS	118.13 ± 25.04	96.04 ± 19.21	0.213
ICU length of stay	28.76 ± 10.05	22.42 ± 13.66	0.460

*APACHE II* Acute Physiology and Chronic Health Evaluation II; *SAPS II* Simplified Acute Physiology Score II, *SOFA* Sequential Organ Failure Assessment score, *LIS* Lung Injury Score, *P/F* PaO_2_/FiO_2_ ratio on the day of diagnosis of ARDS; Values are mean ± SD

The differences between the two conditions are also clearly seen in a life table, Fig. 4e. While the Kaplan–Meier statistic for this table is not quite significant (χ^2^ = 3.65, df = 1, p = 0.056), it is also clear that the improved mortality for the Runx1p66 group begins at about 15 days, and then persists from then on. Another remarkable finding was that 100% of the high p66/p52 ratio sample was still alive at day 100 (not plotted), Additional file 1: Table S1. Thus, correlation of EV_ARDS_ immunoreactivity for Runx1 with survival data indicates that the transient expression of Runx1p66 and a high Runx1p66/p52 ratio provide a survival benefit to ARDS patients.

### Alk5 and Runx1 reside on the same EV_ARDS_ population

We used magnetic separation of Alk5-positive EV_ARDS_ via MagSi-streptavidin beads to address whether Alk5 and Runx1 reside on the same EV_ARDS_ population. Briefly, Alk5-positive EV_ARDS_ were labeled with an anti-Alk5 goat Ab, followed by a biotinylated rabbit anti-goat IgG and MagSi-streptavidin beads. WB analyses of bound and unbound EV_ARDS_ indicated that all EV_ARDS_ immunoreactive to Alk5 possess Runx1 (Fig. 4f), suggesting that a significant sub-set of EV_ARDS_ comprises both Alk5/TβRI receptor and Runx1 transcription factor. However, a small fraction of Runx1-positive EV_ARDS_ does not contain Alk5, Fig. 4f (unbound fractions).

### EV_ARDS_Alk5Runx1p66 stimulate ECs proliferation

**Fig. 5:**
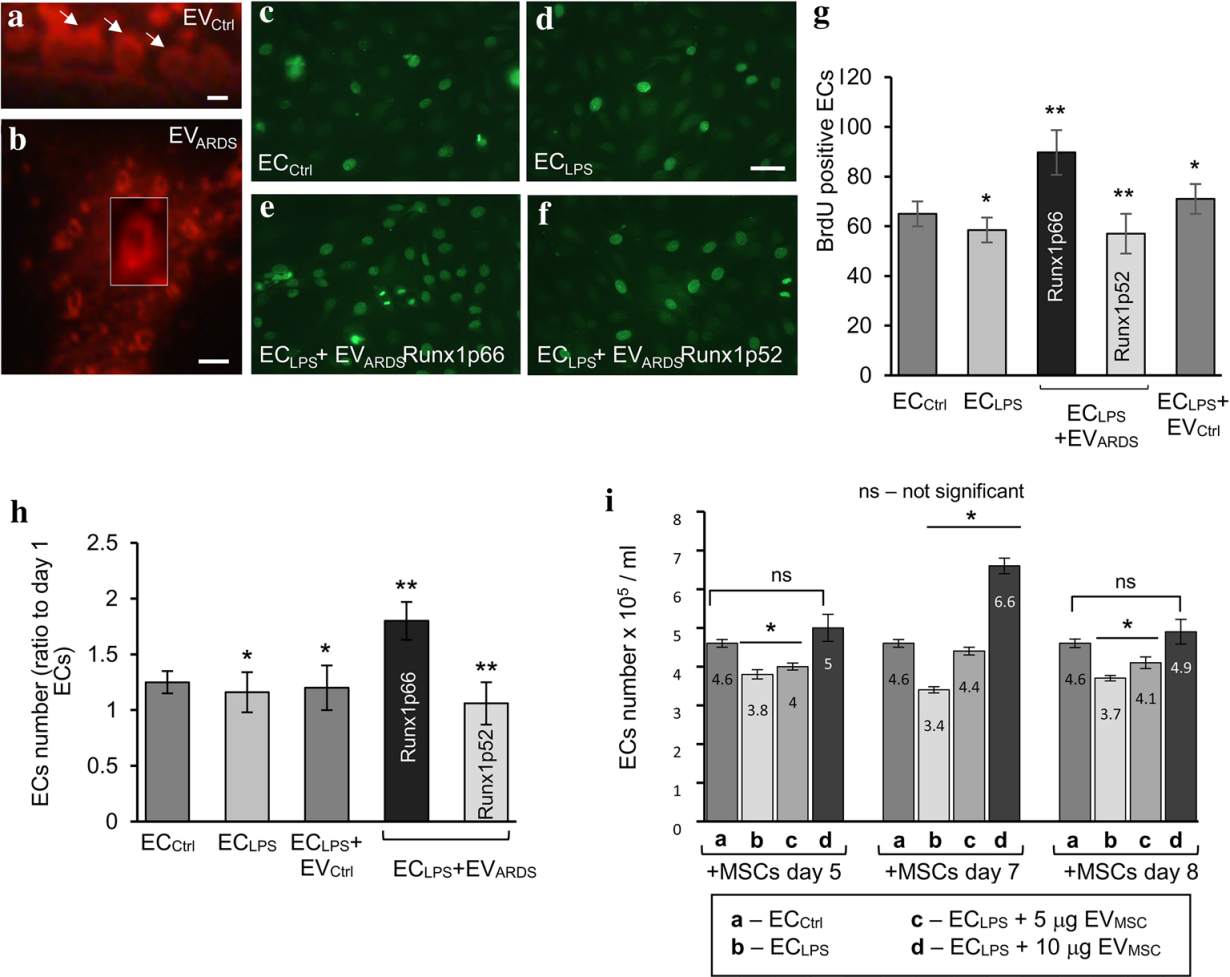
EV_ARDS_Alk5Runx1p66 increase proliferation of LPS-treated ECs. Biotin/neutrAvidin-Alexa Fluor 594 labeling of EVs isolated from a human healthy donor (**a**, EV_Ctrl_) and an ARDS subject (**b**, inset; EV_ARDS_). Arrows in **a** indicate several donut-shaped EV_Ctrl_. Bars: 1 μm (**a**, and **b** inset), 2 μm (**b**). n = 3. BrdU labeling of EC_Ctrl_ (**c**), EC_LPS_ (**d**) and EC_LPS_ exposed to EV_ARDS_Alk5Runx1p66 isolated from the blood of a long-term ARDS survivor (**e**) and EC_LPS_ exposed to EV_ARDS_Alk5Runx1p52 isolated from the blood of a non-surviving ARDS patient (**f**). A dose of 1.6 × 10^3^ EV_ARDS_/1 ml growth medium was used for the micrographs shown. Bars: 50 μm (**c**–**f**). **g** Quantification of BrdU-positive cells. The results are expressed as BrdU-positive ECs/high power field (50 power field images/experimental condition). Data are representative of three different experiments and were normalized per total number of cells counted. Values are shown as mean ± SEM. n = 3; *p < 0.05; **p < 0.01. **h** MTT assay and cell counting of EC_Ctrl_ and EC_LPS_ exposed for 3 days to EV_Ctrl_ and EV_ARDS_, as above (ratio 2:1 EV_ARDS_:EV_Ctrl_). Data are normalized to day 1 EC_Ctrl_. Values are shown as mean ± SEM. n = 3; *p < 0.05; **p < 0.01. **I** EC_Ctrl_ (a bars), EC_LPS_ (b bars), EC_LPS_ + 5 μg EV_MSC_ (c bars) and EC_LPS_ + 10 μg EV_MSC_ (d bars) were counted 3 days post-EV_MSC_ exposure. Briefly, EV_MSC_ released by 3 × 10^6^ MSCs, at day 5, day 7 and day 8, were resuspended in 50 μl sterile PBS; 1 dose of 10 μg/ml protein was found efficient in inducing proliferation of EC_LPS_. All data labels are included for each bar. Values are shown as mean ± SEM. n = 3 experiments (triplicate wells each); *p < 0.01 vs. EC_Ctrl_

First, EV_ARDS_ were visualized by biotin/neutrAvidin–Alexa Fluor 594 labeling, as recently described by us for the EVs isolated from the blood of ITSN-deficient mice [15]. Similar to the mouse EVs, fluorescently labeled human EVs show a continuous, donut-shape (Fig. 5a, arrows; b, inset). As human ARDS lung tissue is deficient in EC proliferation, we next evaluated the biological activity of EV_ARDS_ on cultured human lung ECs proliferation. To mimic the inflammatory EC dysfunction, the ECs were treated with 1 μg/ml LPS for 6 h (EC_LPS_), and then exposed to three doses of EV_ARDS_Alk5Runx1p66 or EV_ARDS_Alk5Runx1p52 [0.8 × 10^3^, 1.6 × 10^3^ (the dose shown in Fig. 5e, f) and 3.2 × 10^3^ per 1 ml growth medium] for 30 h, followed by BrdU cell proliferation assay [24]. ECs with no LPS and no EV_ARDS_ exposure (EC_Ctrl_, Fig. 5c), EC_LPS_ (Fig. 5d) and EC_LPS_ exposed to EV_Ctrl_ at 2:1 (EV_ARDS_:EV_Ctrl_) ratio, according to the in vivo situation (not shown), were used for comparison. Morphometric analyses of the BrdU-positive ECs nuclei (Fig. 5g), indicated that 1 μg/ml LPS for 6 h minimally inhibits ECs proliferation (less than 10%). Exposure of EC_LPS_ to 1.6 × 10^3^ EV_ARDS_Alk5Runx1p66 caused about 38% increase in the BrdU-positive cells compared to EC_LPS_. The EV_ARDS_Alk5Runx1p52 did not induce EC_LPS_ proliferation, regardless of the dose used. Exposure of EC_LPS_ to EV_Ctrl_ (2:1 ratio EV_ARDS_;EV_Ctrl_) increased the number of BrdU-positive cells less than 10%. The proliferative effects of EV_ARDS_Alk5Runx1p66 at a lower or, the higher doses used were less evident. Furthermore, the MTT assay [24] to evaluate the effects of the EV_ARDS_ on LPS-injured ECs (Fig. 5h) biochemically, indicated that 1.6 × 10^3^ EV_ARDS_Alk5Runx1p66/1 ml growth medium at day 3 post-exposure, increase the number of EC_LPS_ by 44%, compared to EC_Ctrl_ (Fig. 5h). EC_LPS_ exposed to EV_Ctrl_ show similar proliferation as EC_LPS_, both slightly lower than EC_Ctrl_. EC_LPS_ exposed to 1.6 × 10^3^ EV_ARDS_Alk5Runx1p52/1 ml growth medium showed some variability in their proliferative effects depending on the EV_ARDS_ sample used; on average however, the EV_ARDS_Alk5Runx1p52 slightly decreased, by less than 8%, the number of the BrdU-positive cells compared to EC_LPS_ (Fig. 5h). Based on these data, we concluded that the under inflammatory conditions, EV_ARDS_Alk5 Runx1p66 stimulate ECs proliferation.

### Runx1p66 accounts for EC proliferation

The proof-of-concept that Runx1p66 isoform accounts for EC proliferation was obtained when EC_LPS_ were exposed to EV_MSC_, isolated from the conditioned media of cultured MSCs (passage 3–4), as described under Methods. EV_MSC_ were isolated at day 7, when Runx1p66 immunoreactivity is detected and at days 5 and 8, with no Runx1p66 is detected; two EV_MSC_ doses were used: 5 μg/ml (low dose) and 10 μg/ml (high dose). The quantitative assessment indicated that 10 μg/ml EV_MSC_ increased proliferation of EC_LPS_ at day 7 by 40%, compared to EC_Ctrl_, Fig. 5I (bar d vs. bar a).

Minimal EC proliferation, with no statistical significance was noted at days 5 and 8, for the high EV_MSC_ dose. No EC proliferation was noticed for the low EV_MSC_ dose, regardless of Runx1p66 content; the finding is consistent with an EV_MSC_ basal threshold for inducing proliferation, as already reported by us [15] and with the beneficial effects of the high Runx1p52/p66 ratio. The data support the causal impact of Runx1p66 expression on EC proliferation.

### EV_ARDS_Alk5Runx1p66 improve the interendothelial junctional integrity of LPS-treated ECs

**Fig. 6:**
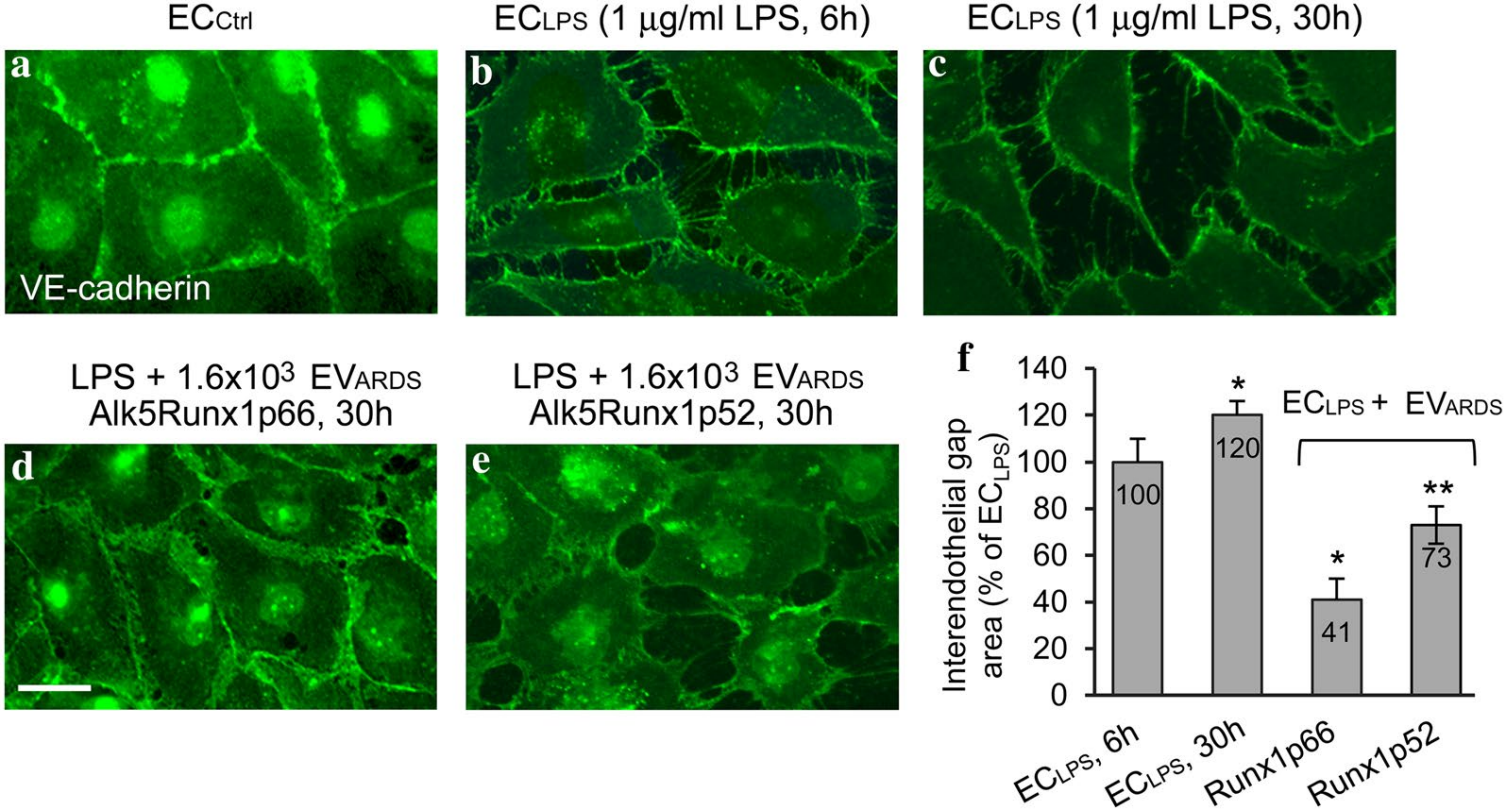
EV_ARDS_Alk5Runx1p66 improve the interendothelial junctional integrity of LPS-treated ECs. Representative VE-cadherin/Alexa Fluor 488 staining of EC_Ctrl_ (**a**), ECs treated with 1 μg/ml LPS (EC_LPS_) for 6 h (**b**) and 30 h (**c**); EC_LPS_ (6 h) were treated for 30 h with either (**d**) 1.6 × 10^3^ EV_ARDS_Runx1p66/1 ml growth media or (**e**) 1.6 × 10^3^ EV_ARDS_Runx1p52/1 ml growth media (non-surviving ARDS patient). Bars: 15 μm (**a**–**e**). **f** Morphometric analysis of the interendothelial gap area. All data labels are included for each bar. n = 3 EVs preparations/experimental condition, in three independent experiments; *p < 0.01; p** < 0.05

As the integrity of the interendothelial junctions (IEJs) is vital for barrier function and edema resolution, we investigated the biological activity of freshly isolated EV_ARDS_ in mitigating the LPS effects on IEJs. Cultured ECs treated with 1 μg/ml LPS for 6 h, as above followed by 30 h exposure to three doses (0.8 × 10^3^, 1.6 × 10^3^ and 3.2 × 10^3^ per 1 ml growth medium) of EV_ARDS_Alk5Runx1p66, were subjected to VE-cadherin Ab/Alexa Fluor488 immunocytochemistry. The integrity of IEJs in EC_LPS_ treated with 1.6 × 10^3^ EV_ARDS_Alk5Runx1p66 (Fig. 6d) was improved compared to EC_LPS_ with no EV_ARDS_Alk5Runx1p66 exposure (Fig. 6b, c) or exposed to an equal dose of EV_ARDS_Alk5Runx1p52 of a non-surviving ARDS patient (Fig. 6e). Figure 6a shows for comparison the IEJs in EC_Ctrl_. Interendothelial gaps are significantly larger at 30 h post-LPS exposure without any EV_ARDS_ treatment (Fig. 6c) compared to 6 h LPS time point (Fig. 6b). Quantitative assessment of intercellular gaps (NIH ImageJ), shows that EV_ARDS_ bearing Runx1p66 reduced significantly, more than 60% the interendothelial gap area in LPS-treated ECs, while the EV_ARDS_ with no Runx1p66 by only 27%, Fig. 6f. The surface of the interendothelial gaps at 30 h post-LPS is 40% greater compared to 6 h LPS time point with no EV_ARDS_ exposure. The effects of the lower and the higher EV_ARDS_ doses were less noticeable.

### EV_ARDS_Alk5Runxp66 decrease lung histological severity in LPS-treated mice

**Fig. 7:**
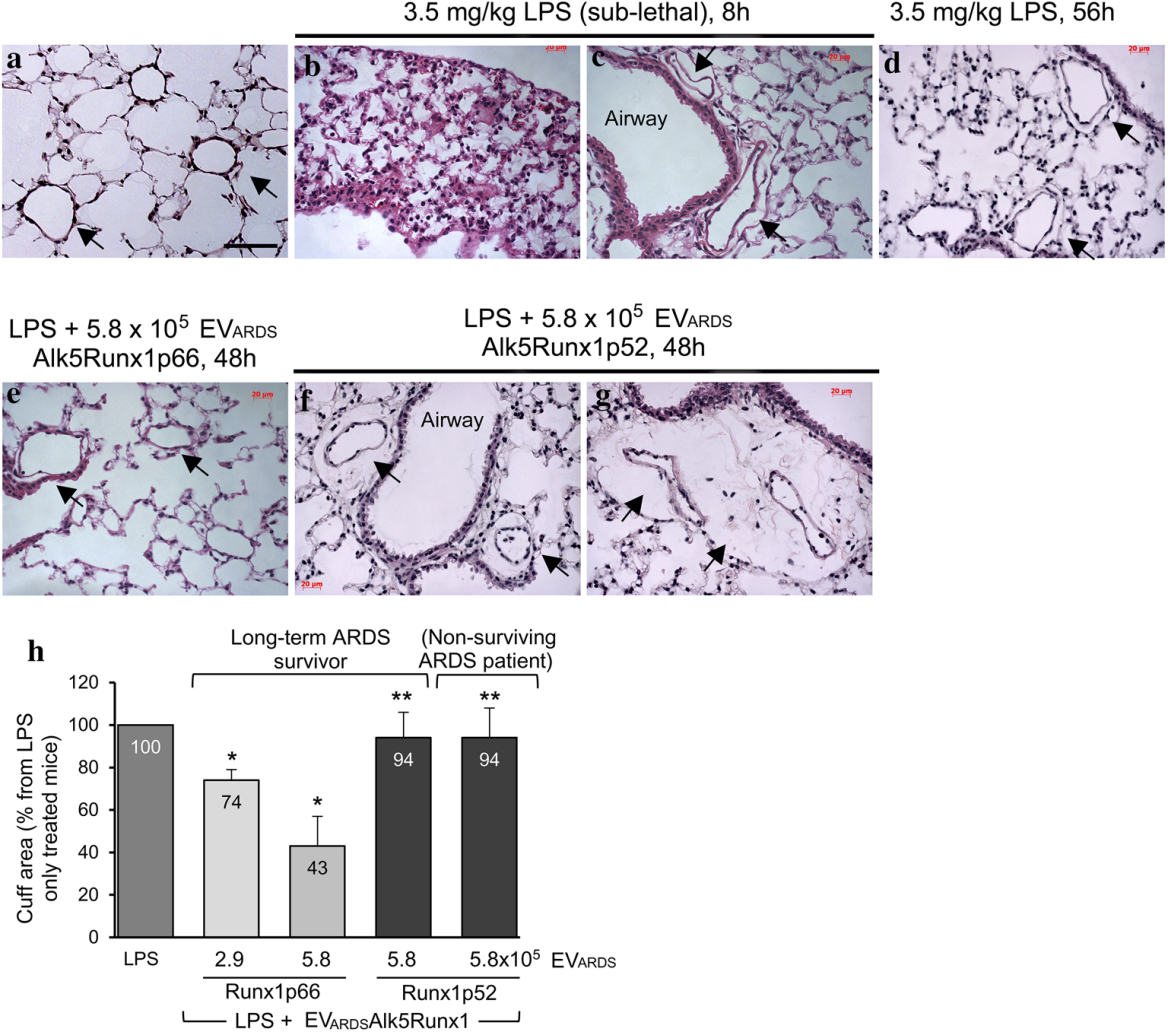
EV_ARDS_Alk5Runx1p66 decrease lung histological severity and restore lung endothelial permeability in LPS-treated mice. Representative H&E of lung sections illustrates the extent of perivascular cuffs (arrows) and thickening of the interstitium in mice treated with LPS only (**b**, **c**, **d**), EV_ARDS_Alk5Runx1p66 (**e**), EV_ARDS_Alk5Runx1p52 isolated from the blood of a long-term ARDS survivor, week 1 in ICU, when the EV_ARDS_ do not show Runx1p66 immunoreactivity (**f**) and EV_ARDS_Alk5Runx1p52 of a non-surviving ARDS (**g**). **a** Comparison the H&E staining of a control, untreated mouse lung section. **h** Morphometric analyses of perivascular cuff area using the NIH ImageJ software; only small and medium-sized (20 ≥ diameter ≤ 100 μm) blood vessels were included. All data labels are included for each bar. Bar: 50 μm (**a**–**g**). n = 3 different EV_ARDS_ preparations in three independent experiments; *p < 0.01; **p < 0.05

Lung injury was induced in mice by intraperitoneal delivery of 3.5 mg/kg LPS (sub-lethal dose) as described in [6]; 8 h post-LPS treatment, mice were injected retro-orbitally with two EV_ARDS_ doses: 2.9 × 10^5^ and 5.8 × 10^5^ (dose shown in Fig. 7) in 100 μl sterile PBS. Assessment of lung pathology on H&E stained mouse lung sections, 48 h after EV_ARDS_Alk5Runx1p66 administration shows reduction of inflammatory cells, decreased perivascular cuffing and less thickening of the interstitium (Fig. 7e) compared to LPS-only treated mice (Fig. 7b–d). Lung histological severity is not improved by EV_ARDS_Alk5Runx1p52 (EV_ARDS_ of the same patient in week 1 in ICU, when no Runx1p66 immunoreactivity is detected), Fig. 7f, or EV_ARDS_ a non-surviving patient (week 2 ICU), Fig. 7g. The 48 h time point post-EV_ARDS_ has been selected as mice need at least 72 h for recovery after sub-lethal LPS, with no other treatment [40]. The benefit of the low EV_ARDS_Alk5Runx1p66 dose was less evident (not shown). The lung histology of mice treated with LPS only and mice treated with LPS followed by EV_Ctrl_ display no detectable differences at this time point (not shown). Mice injected with PBS served as controls (Fig. 7a). Quantitative assessment of perivascular cuffs, sign of a compromised endothelial barrier indicates ~ 60% reduction in EV_ARDS_Alk5Runx1p66-treated mice, when the high dose has been used, compared to LPS-treated mice (Fig. 7h). The beneficial effects of the low EV_ARDS_Alk5Runx1p66 dose were less visible (Fig. 7h). Mice treated with EV_ARDS_Alk5Runx1p52, regardless of the source of the EV_ARDS_ preparation—non-surviving ARDS subjects or long-term survivors—show no decline in the perivascular cuffing (Fig. 7h). These data strongly suggest that despite the complex biochemical makeup of EV_ARDS_, Runx1p66 contributes significantly to improve the junctional integrity and reduce perivascular cuffing of LPS-injured ECs.

## Discussion

In the current study, we have identified in the blood of long-term ARDS survivors a subset of EV_ARDS_ with MSC origin and different phenotype compared to the circulatory EV_ARDS_ of non-surviving patients. To our knowledge, the presence in the blood of long-term ARDS survivors of a distinct population of circulatory EV_ARDS_ with MSC-origin and different biochemical makeup compared to the EV_ARDS_ of non-surviving patients has not been reported so far. Similar to our observations however, limited studies reported a protective role of leukocyte-derived EVs with an increased count in the blood and bronchoalveolar lavage of ARDS patients [41]. More recently, Shaver et al. [42], found a strong association between the lower levels of circulatory EVs and the development of ARDS in patients with sepsis. Nonetheless, the biochemical makeup or mechanism(s) involved remain unknown. We show now that a subset of the EV_ARDS_ with MSC origin and increased count comprises the widely expressed TβR1/Alk5 and the Runx1 transcription factor. The protein expression pattern of two Runx1 isoforms during the first month in the ICU appears critical for the ARDS outcome: the p52 isoform shows a continuous expression, while the p66 isoform is short-lived. Significantly, a high ratio Runx1p66*/*p52 was associated with survival. We have also found that this difference in the temporal expression pattern of Runx1 isoforms is a characteristic of cultured human bone marrow derived MSCs, as well, consistent with the recent similar reports for cells with non-hematopoietic lineage [43].

Previous studies have shown that the MSCs decrease the severity and even improve survival in various animal models of ALI/ARDS [44–47]. MSCs produce a wide variety of molecules including hematopoietic and angiogenic factors as well as chemokines and generally, render their effects by immunomodulation [48]. Recently, Zhu and colleagues showed that MSC-derived EVs are therapeutically effective following *Escherichia coli* endotoxin-induced ALI [49], at least in part due to the expression of keratinocyte growth factor mRNA transferred to epithelium by these EVs. To date, no study has looked at the role of EV_MSC_ in endothelial recovery in ARDS. Loss of endothelial barrier function is one of the key events for the development of ARDS. It is both necessary and sufficient for the pathogenesis of ARDS regardless of epithelial damage [50, 51]. As shown by multiple studies, MSCs improve both endothelial and epithelial permeability and function [48]. Also, since every patient’ response varies to similar injury (e.g. sepsis), the severity of ARDS as well as clinical course, vary. This variability can be in part played by the presence of endogenous MSCs and their mediators that are delivered to injured ECs by EVs.

Similar to the ITSN-deficient mouse studies, the sub-population of human MSC-derived EV_ARDS_ are immunoreactive to both TβR1 and TβRII, a strong indication that these EV_ARDS_ are equipped with “ready-to-signal” TβR1/TβRII heteromeric complexes [52]. Our recent work demonstrated a functional relationship between the intercellular transfer of Alk5 by circulatory EVs and ECs proliferation via a novel molecular mechanism for TGFβ/Alk5-dependent Erk1/2 kinase signaling [15]. We have shown that ITSN deficiency leading to non-productive assembly of the Alk5-Smad-SARA (Smad anchor for receptor activation, also known as ZFYVE9) signaling complex and preferential formation of the Alk5–mSos–Grb2 complex accounts for Erk1/2 activation downstream of Alk5 and proliferation of pulmonary ECs. Thus, after the interaction of EV_ARDS_ with the injured, ITSN-deficient ECs, the TGF-β/Alk5 signaling switches from Smad2,3 to Erk1/2 MAPK pathway and downstream Cdk6 activation leading to the proliferation of ECs and microvascular remodeling [15, 53]. Runx1 is a target for both Cdk6 and Erk1/2 [35, 54], and it seems that in inflammatory settings associated with ARDS, the TGFβ/Alk5-dependent Erk1/2 kinase activation, Cdk6 regulation and Runx1 transcriptional activity are responsible for ARDS-associated microvascular remodeling and lung tissue repair.

Runx1, a member of runt-related transcription factors, is critically necessary for angiogenesis, T-cell and B-cell maturation [55, 56] and regulation of the cell cycle [56]. It has been reported that Runx1 plays a critical role in the mouse lung inflammation following LPS-induced injury [57]. Increased respiratory distress, inflammation, and pro-inflammatory cytokine were observed in the Runx1-deleted mice after pulmonary LPS exposure; Runx1 deletion was associated with the activation of NFκ-B in respiratory epithelial cells [57].

In our studies, we found that EV_ARDS_ carry two Runx1 isoforms, p52 and p66. As shown in Table 3, the high ratio Runx1p66/p52 was associated with increased survival (likelihood ratio 5.218). Age, sex, SAPS II, APACHE II, SOFA, LIS, P/F ratio at the onset of ARDS or ICU length of stay cannot explain the difference. Patients with Runx1p66 isoform and a high ratio p66/p52 in their EV_ARDS_ seemed to have a higher survival as compared to patients who did not. Through this observation, it is possible that we can use the expression pattern of Runx1 isoforms as a reliable circulatory biomarker of ARDS activity.

Interestingly, our observation that EV_ARDS_Runx1p66 might be needed only when the endogenous MSCs and lung ability to repair are not sufficient, suggests that in some long-term survivors lung resident stem cells may be involved in achieving the epithelial repair in ARDS. In fact, limited studies reported the presence of a specialized lung resident stem cell niche where type II pneumocytes function hand-in-hand with the mesenchymal stromal cells to achieve the epithelial repair following injury; it appears that the lung resident stem cell migrate from their in-tissue niche into the alveolar space and are abundant and recoverable from the bronchoalveolar lavage fluid [36].

We also observed that expression of Runx1p52 in long-term survivors gradually increases from day 1 to 37 of ICU stay (Fig. 4b). As Runx1 attenuation induces myofibroblast differentiation [34], it appears that increased expression of Runx1p52 functions to protect against excessive/pathological fibroproliferation. Thus, Runx1p52 isoform may be a novel target for therapeutic interventions to prevent pathological fibroproliferation in ARDS and a new avenue for treating severe ARDS.

Our study is first of its kind which proposes an Alk5/Runx1-mediated mechanism by which bone marrow-derived MSCs via their released EVs rescue and repair pulmonary microvascular cellular injury in ARDS. Exposure of LPS-injured human lung ECs to EV_ARDS_Alk5Runx1p66 increases proliferation and improve junctional integrity; moreover, in LPS-treated mice, these EV_ARDS_ bearing the Runx1p66 isoform, decrease lung histological severity. By contrast, exposure to EV_ARDS_Alk5Runx1p52 regardless of their long-term survivors or non-surviving patients source cannot induce endothelial recovery, strongly supporting the concept that Runx1p66 accounts for the beneficial effects of the EV_ARDS_Alk5Runx1p66, and thus for the improved outcome of the long-term ARDS survivors. As the EV_ARDS_Alk5Runx1p52 show no therapeutic benefit on LPS-treated ECs and LPS-mouse model of ALI, it appears that even if the EV_ARDS_ possess the TβR1/TβRII heteromeric complexes, the lack of the Runx1p66 isoform is critical for ARDS resolution and survival.

Though it is a robust study, we performed this translational approach in a relatively small number of ARDS patients, with a clear signal for survival in patients with EV_ARDS_Alk5Runx1p66; however, due to small sample size and clinical heterogeneity, our finding should be confirmed in a larger cohort of patients. Our cohort included 33 ARDS patients, 29 with direct lung injury caused by pneumonia and 4 with indirect lung injury, caused by sepsis. While there may be significant overlap between direct and indirect ARDS in humans [58], the diverse treatment/medication received, concurrent illness, mechanical ventilation, use of the extracorporeal membrane oxygenation, active inflammation, etc., and the small study population without possibility of multivariable analyses may also contribute to the difference in ARDS outcome.

One other limitation of the study is the use of healthy subjects as controls. However, the contrast between the lower levels of circulating EVs in healthy conditions by comparison to disease state has been extensively reported in the literature [7, 9, 11]. Despite these limitations, this study identifies a specific subset of EV_ARDS_Alk5Runx1p66 of MSC origin that portends a favorable prognosis for ARDS patients. Thus, Runx1p66-expressing EVs derived from MSCs cultures, have the potential of providing rapid, effective and clinically safe therapeutic approaches, and may translate into a novel ‘paradigm shift’ strategy to efficiently treat ARDS and promote survival. As the MSCs phenotype is different depending on the growth stages, the observation needs to be considered when the therapeutic efficiency of the MSCs is investigated.

In sum, studies to evaluate whether or not the EV_ARDS_Alk5Runx1p66 have a broad therapeutic effect beyond the LPS injury and to validate the finding in a larger cohort of ARDS patients will help to establish the expression pattern of Runx1 isoforms not only as a reliable circulatory biomarker of ARDS activity, but also as a novel determinant of the molecular mechanism for lung tissue repair and recovery after severe injury.

## Conclusions

The expression pattern of Runx1 isoforms might be a reliable circulatory biomarker of ARDS activity and a novel determinant of the molecular mechanism for lung tissue repair and recovery after severe injury.

## Additional file

**Additional file 1: Table S1.** Human Subjects. Detailed Clinical Data Set.

## Abbreviations

ALI: acute lung injury
APACHE II: Acute Physiology and Chronic Health Evaluation II
APC: allophycocyanin
ARDS: acute respiratory distress syndrome
BCA: bicinchoninic acid
BrdU: bromodeoxyuridine
ECs: endothelial cells
EC_LPS_: lipopolysaccharide (LPS)-treated ECs
ECMO: extracorporeal membrane oxygenation
EVs: extracellular vesicles
EV_MSC_: mesenchymal stem cells-derived extracellular vesicles
EV_ARDS_: extracellular vesicles isolated from the blood of ARDS patients
EV_Ctrl_: extracellular vesicles isolated from the blood of control subjects
H&E: hematoxylin & eosin
ICU: intensive care unit
IHC: immunohistochemistry
ITSN: intersectin-1short
LIS: Lung Injury Score
LPS: lipopolysaccharide
MSCs: mesenchymal stem cells
MTT: 3-(4,5-dimethylthiazol-2-yl)-2,5-diphenyl tetrazolium bromide
ND-Ctrl: non-disease controls
TβRI: transforming growth factor receptor i
TβRII: transforming growth factor receptor ii
PBS: phosphate buffered saline
PE: phycoerythrin
P/F: PaO_2_/FiO_2_
RT: room temperature
RUMC: Rush University Medical Center
Runx1p52: Runx 1 p52 isoform
Runx1p66: Runx 1 p66 isoform
SAPS II: Simplified Acute Physiology Score II
SOFA: Sequential Organ Failure Assessment
