# Supplementary material for: Alk5/Runx1 signaling mediated by extracellular vesicles promotes vascular repair in acute respiratory distress syndrome
Source: Clin Transl Med. 2018 Jun 22;7:19. doi: 10.1186/s40169-018-0197-2 (PMC6013417; doi:10.1186/s40169-018-0197-2)
Supplement: Supplementary file 1 — Additional file 1: Table S1. Human Subjects. Detailed Clinical Data Set. [file 40169_2018_197_MOESM1_ESM.docx]

| Sample # | Age | Sex (M/F)/race | SAPS II  Score | APACHE II  Score | SOFA  Score | Lung Injury Score | P/F  ratio |  | Runx1^p66^  expression  p66/p52  ratio |  | ICU length of stay (days) | Mortality | Ventilator free days | ECMO | Cause of ARDS |
| --- | --- | --- | --- | --- | --- | --- | --- | --- | --- | --- | --- | --- | --- | --- | --- |
| S2 | 23 | Male/White (non-Hispanic) | 45 | 15 | 15 | 2.25 | 68 | Yes | >.5 |  | 50 | Alive (at 100 days) | None | Yes | Streptococcal pneumonia |
| S3 | 23 | Male/White (non-Hispanic) | 45 | 27 | 12 | 3.75 | 76 | No | --- |  | 14 | Alive (at 100 days)) | 7 | No | Aspiration pneumonia |
| S4 | 20 | Female/White (non-Hispanic) | 28 | 15 | 13 | 2.5 | 115 | Yes | >.5 |  | 27 | Alive (at 100 days) | 28 | No | Multilobar pneumonia |
| S5 | 65 | Male/Black | 68 | 34 | 14 | 3.75 | 77 | Yes | >.5 |  | 3 | Deceased* | NA | Yes | Aspiration pneumonia |
| S6 | 46 | Female/Hispanic-white | 45 | 19 | 10 | 3.5 | 90 | Yes | >.5 |  | 32 | Alive (at 100 days) | 8 | Yes | Influenza B and MRSA pneumonia |
| S7 | 58 | Male/White (non-Hispanic) | 44 | 26 | 13 | 3.75 | 132.5 | No | --- |  | 13 | Deceased | NA | No | Intra-abdominal sepsis |
| S8 | 54 | Female/Black | 68 | 34 | 18 | 3 | 49 | No | --- |  | 7 | Deceased | NA | No | Intra-abdominal sepsis |
| S9 | 62 | Male/White Hispanic | 48 | 21 | 7 | .75 | 95 | No | --- |  | 13 | Alive (at 100 days) | 20 | No | Community acquired pneumonia |
| S10 | 30 | Male/Black | 44 | 29 | 10 | 2 | 175 | Yes | >.5 |  | 24 | Alive (at 100 days) | None | No | Secondary to sepsis |
| S11 | 31 | Female/Black | 86 | 43 | 21 | 3.75 | 58 | Yes | <.5 |  | 12 | Deceased | NA | No | Secondary to sepsis/toxic megacolon |
| S12 | 51 | Male/Black | 45 | 16 | 11 | 3.5 | 91 | yes | <.5 |  | 87 | Deceased | NA | Yes | Healthcare associated pneumonia |
| S13 | 49 | Female/White non-Hispanic | 37 | 26 | 6 | 3 | 162 | yes | >.5 |  | 12 | Alive (at 100 days) | 14 | No | Aspiration |
| S14 | 49 | Male White non-Hispanic | 71 | 33 | 10 | 4 | 91 | yes | >.5 |  | 12 | Deceased** | NA | No | Community acquired pneumonia |
| S15 | 48 | Female/white non-Hispanic | 55 | 28 | 11 | 3.75 | 86 | yes | <.5 |  | 54 | Deceased | NA | No | Influenza |
| S17 | 67 | Feale/White Hispanic | 36 | 19 | 6 | 1.5 | 232 | yes | >.5 |  | 26 | Alive (at 100 days) | 13 | No | Community acquired pneumonia - human metapneumovirus |
| S18 | 39 | Male/White Hispanic | 46 | 24 | 10 | 3 | 167 | yes | >.5 |  | 98 | Alive (at 100 days) | 10 | Yes | Viral pneumonia |
| S19 | 29 | Female/Black | 44 | 23 | 13 | 3.5 | 170 | No | --- |  | 6 | Alive (at 100 days) | 10 | No | Influenza |
| S20 | 76 | Male/White (non-Hispanic) | 52 | 28 | 14 | 3 | 73 | No | --- |  | 28 | Deceased | NA | Yes | Aspiration pneumonia |
| S21 | 40 | Male/Middle Eastern | 54 | 21 | 9 | 3.3 | 73 |  | ND |  | 91 | Alive (at 100 days) | None | No | Influenza |
| S22 | 71 | Male/White non-Hispanic | 41 | 17 | 8 | 2.8 | 125 | No | --- |  | 10 | Deceased | NA | No | Healthcare associated pneumonia |
| S23 | 41 | Female/Hispanic | 59 | 17 | 15 | 2.5 | 111 | No | --- |  | 11 | Alive (at 100 days) | 5 | No | Small bowel obstruction post exploratory laparotomy |
| S24 | 30 | Male/Black | 28 | 17 | 7 | 3.3 | 133.7 |  | ND |  | 22 | Alive (at 100 days) | 10 | No | Klebsiella pneumonia |
| S25 | 29 | Female/Black | 61 | 24 | 11 | 2.8 | 115 | Yes | >.5 |  | 33 | Alive (at 100 days) | None | No | Pneumocystis Jiroveci pneumonia |
| S26 | 46 | Male/Black | 38 | 21 | 13 | 3 | 76 | Yes | >.5 |  | 20 | Alive (at 100 days) | None | No | Aspiration |
| S27 | 59 | Male/White non-Hispanic | 53 | 26 | 14 | 3 | 86 | No | --- |  | 14 | Alive (at 100 days) | 10 | No | Healthcare associated pneumonia |
| S28 | 51 | Female/White | 53 | 30 | 13 | 3.5 | 70 | Yes | >.5 |  | 30 | Alive (at 100 days) | 5 | No | Influenza |
| S29 | 45 | Female/White | 30 | 17 | 6 | 2.25 | 144 |  | ND |  | 8 | Alive (at 100 days) | 6 | No | Influenza |
| S30 | 37 | Female/Black | 20 | 12 | 7 | 3.25 | 75 |  | ND |  | 11 | Alive (at 100 days) | 11 | No | Organizing pneumonia |
| S31 | 37 | Female/Black | 34 | 22 | 8 | 2.8 | 115 | Yes | >.5 |  | 40+ | Alive (at 100 days) | 6 | Yes | Pancreatitis |
| S32 | 50 | Female/Hispanic | 34 | 12 | 7 | 2 | 210 | Yes | >.5 |  | 14 -15 | Alive | None | No | Healthcare associated pneumonia |
| S33 | 47 | Female/Hispanic | 29 | 17 | 10 | 3 | 113.3 | Yes | >.5 |  | 30+ | Alive (at 100 days) | 17 | Yes | Community acquired pneumonia |
| S34 | 36 | Female/Black | 67 | 25 | 19 | 3.5 | 57 | Yes | >.5 |  | 23+ | Alive (at 100 days) | 47 | Yes | Aspiration |
| S35 | 26 | Male/Hispanic | 67 | 26 | 11 | 3.5 | 75 | Yes | >.5 |  | 14 | Deceased** | NA | No | Pneumonia, AIDS |
| Total  33 | 44.1 ± 14.5 | 16M/18F | 47.7  ±  14.6 | 23.3  ±  6.8 | 11.3  ±  3.7 | 11.3  ±  3.7 | 108.2 ±  44.3 |  |  |  | 28.1  ±  24.7 | 29.4 % Mortality |  |  |  |

**Additional file 1: Table 1S. Human Subjects. Detailed Clinical Data Set**

We used un-used blood collected from ARDS patients and healthy controls. Blood samples were provided by the Rush Medical Laboratory Core (IRB# 14030705-IRB01 for investigational use of un-used diseased blood samples that would otherwise be discarded). We have used blood collected from 33 ARDS patients, 16 males and 17 females. Patients enrolled in the study were identified within 24h of diagnosis with following inclusion and exclusion criteria:

- Inclusion criteria: Age > 18 years and met “The Berlin Definition of ARDS” [ARDS Definition Task Force *et al*. Acute respiratory distress syndrome: the Berlin Definition. *JAMA* **307**, 2526-2533 (2012)].
- Exclusion criteria: Age < 18 years, isolated left heart failure and active malignancy. We excluded patients who received immunosuppressants or chemotherapy during the period of ARDS/hospitalization.

In regard to the Racial/Ethnic categories: the cohort of ARDS patients included:

1. ARDS patients Not Hispanic or Latino:
   - - 12 Black or African American, 5 males and 7 females;
     - 10 white, 6 males and 4 females;
     - 1 male of Unknown or Not Reported Racial Category;
2. ARDS patients Hispanic or Latino,
   - - 9 White, 7 females and 2 males;
     - 1 Hispanic, 1 male;

Abbreviations: APACHE- Acute Physiology and Chronic Health Evaluation (clinical scoring system to classify the severity of the disease); SAPS- Simplified Acute Physiology Score (Score to calculate the probability of hospital mortality); SOFA- Sequential Organ Failure Assessment (score to describe the degree of organ dysfunction); Lung Injury Score – use to describe the presence and extent of pulmonary damage; a score >2.5 is used for the definition of ARDS. P/F - PaO_2_/FIO_2._ ECMO - extracorporeal membrane oxygenation.

Four out of nine ARDS blood samples were used for flow cytometry analyses only; for these 4 samples, Runx1 immunoreactivity has not been determined. Blood collected from three healthy volunteers served as controls.
